# Supplementary material for: Selective observation of semi-rigid non-core residues in dynamically complex mutant huntingtin protein fibrils
Source: J Struct Biol X. 2022 Nov 11;6:100077. doi: 10.1016/j.yjsbx.2022.100077 (PMC9677204; doi:10.1016/j.yjsbx.2022.100077)
Supplement: Supplementary data 1 [file mmc1.pdf]

Supplementary Material

Selective observation of semi-rigid non-core residues in dynamically complex mutant huntingtin protein fibrils

Irina Matlahov [a], Jennifer C. Boatz [b,1] and Patrick C. A. van der Wel [a,b,*]

[a] Zernike Institute for Advanced Materials, University of Groningen, Groningen, The Netherlands
[b] Department of Structural Biology, University of Pittsburgh School of Medicine, Pittsburgh, PA, USA

* Corresponding author: p.c.a.van.der.wel@rug.nl, phone +31 (0) 50 363 26 83; Nijenborgh 4, 9747AG Groningen, The Netherlands

[1] Present address: Division of Pulmonary and Critical Care Medicine, University of Pittsburgh School of Medicine, Pittsburgh, PA, USA

Table S1. Experimental parameters of MAS NMR experiments. Abbreviations: NS, number of scans (per t_1 point for 2Ds); Temp., temperature; MAS, magic angle spinning rate; RD, recycle delay; Mixing, ^{13}C-^{13}C or ^1H-^1H mixing time (ms); t_1 evol., maximum t_1 evolution time expressed in number of t_1 points (real+imaginary) x t_1 increment time; CP time, cross polarization contact time (in ms).

Experiment	Figure	NS	MAS	RD	t_1 evol.	Mixing	CP time	Temp
			kHz	s	ms	ms	ms	K
1D ^{13}C CP	2A,2C,S1A, S2A,	256	10	3	NA	NA	1	277
1D ^{13}C CP	2A,S1A, S2B	256	10	3	NA	NA	1	270
1D ^{13}C CP	2A,2C,S1A, S2C	256	10	3	NA	NA	1	263
1D ^{13}C CP	2A,S1A, S2D	256	10	3	NA	NA	1	256
1D ^{13}C DE	2B,S1B	256	10	3	NA	NA	NA	277
1D ^{13}C DE	2B,S1B	256	10	3	NA	NA	NA	270
1D ^{13}C DE	2B,S1B	256	10	3	NA	NA	NA	263
1D ^{13}C DE	2B,S1B	256	10	3	NA	NA	NA	256
1D ^{13}C INEPT	S1C	256	8.33	3	NA	NA	NA	277
1D ^{13}C INEPT	S1C	256	8.33	3	NA	NA	NA	270
1D ^{13}C INEPT	S1C	256	8.33	3	NA	NA	NA	263
1D ^{13}C INEPT	S1C	256	8.33	3	NA	NA	NA	256
1D ^1H	2D,2E	8	10	2.8	NA	NA	NA	277
1D ^1H	2D,2E	8	10	2.8	NA	NA	NA	270
1D ^1H	2D,2E	8	10	2.8	NA	NA	NA	263
1D ^1H	2D,2E	8	10	2.8	NA	NA		256
2D DARR	3A,S4,7A	80	10	2.8	9.9 (624x32μs)	25	1	277
2D DARR	3B	32	10	2.8	5.6 (414x27.025μs)	25	0.75	256
2D PDSD	S3	32	10	2.8	5.6 (414x27.025μs)	500	0.75	256
2D DIPSHIFT-DARR	7B,S4	32	10	2.8	8.9 (660x27.025μs)	25	0.75	277
1D DIPSHIFT	5B,5D	128	10	3	NA	vary	1	277
1D DIPSHIFT	5C,5E	128	10	3	NA	vary	1	256
2D NCO	4A,4C	128	13	2.8	15 (74x410)	NA	1.5, 4	277
2D NCO	4B,4C	192	10	2.8	11.9 (174x137)	NA	0.8, 4	256
2D NCA	4A	128	13	2.8	15 (74x410)	NA	1.5, 4	277
2D NCA	4B	192	10	2.8	11.9 (174x137)	NA	0.8, 4	256

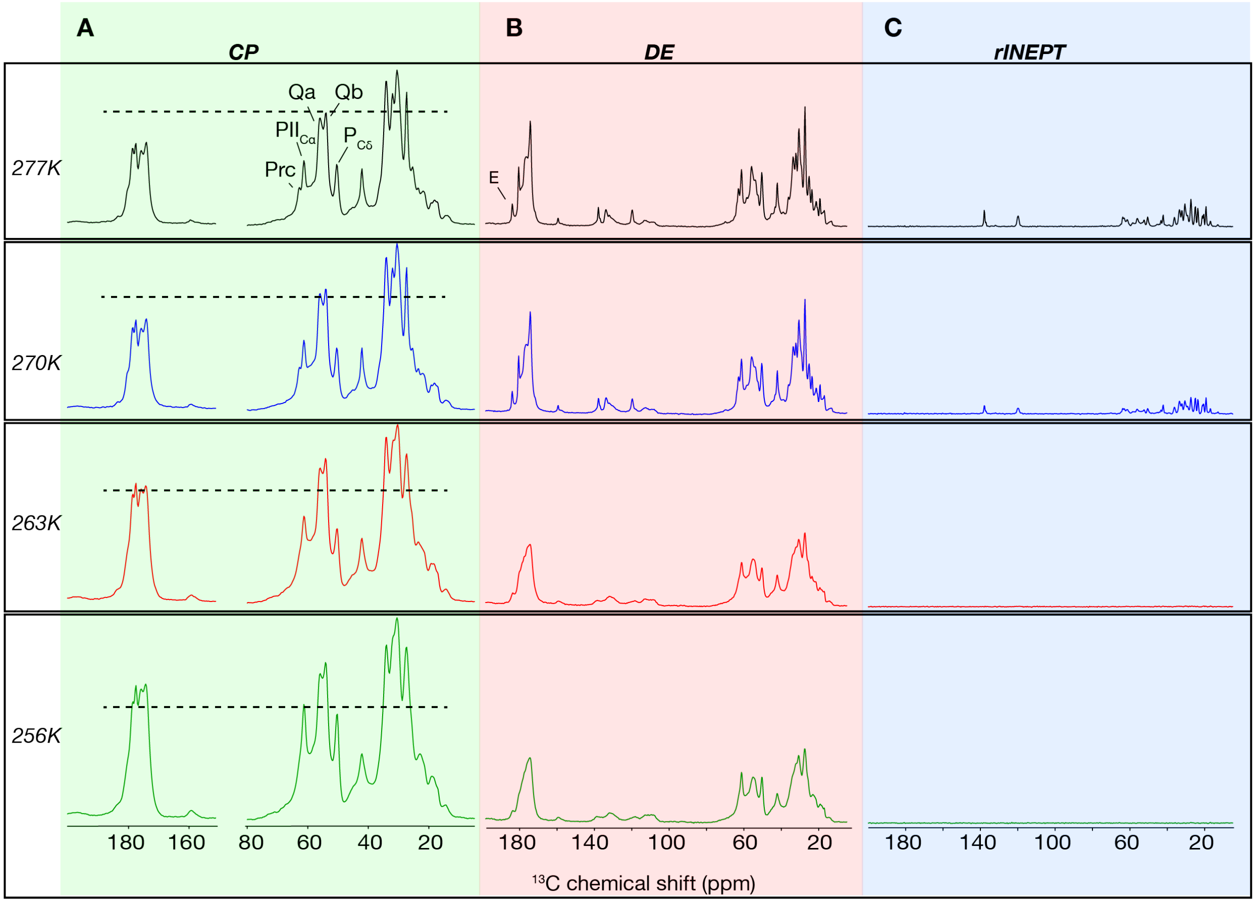

Figure S1. Variable temperature 1D MAS ssNMR of Q44-HttEx1 fibrils. (A) 1D ^{13}C CP-MAS ssNMR at 277, 270, 263 and 256K, reflecting signals of rigid or immobilized parts of the fibrils. (B) 1D ^{13}C direct excitation (DE) MAS ssNMR showing signals of both rigid and mobile residues. (C) 1D ^{13}C refocused INEPT showing highly mobile residues. All data acquired at 600 MHz (^1H) and 10kHz MAS rates. Assignments are based on ref. (Lin et al., 2017).

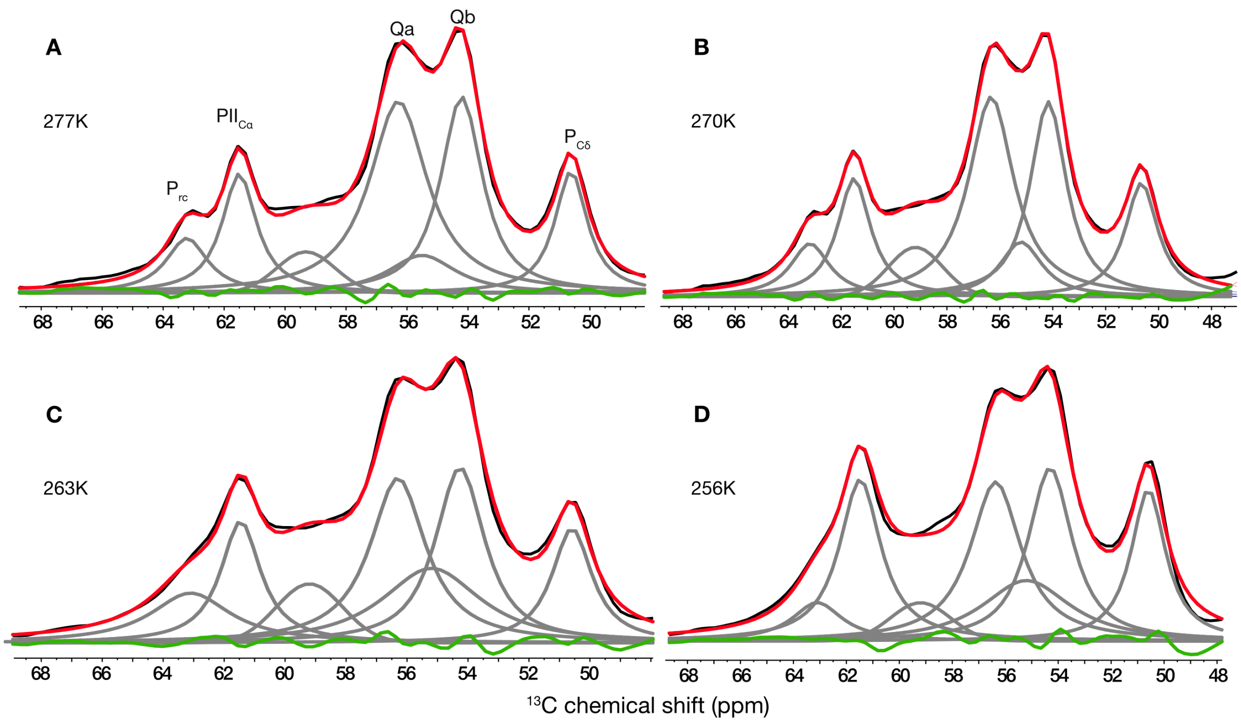

Figure S2. Zoomed region of the 1D ^{13}C CP spectra with deconvoluted glutamine and proline signals. (A) 277K (B) 270K (C) 263K (D) 256K. Black line indicates experimental spectrum. Grey signals indicate deconvolution. Red spectrum is overall spectrum after deconvolution is applied. Green line indicates difference between experimental (black) and the spectrum after deconvolution (red).

Table S2. Signal line widths of glutamine and proline peaks at different temperatures. The uncertainty of linewidths is ±0.1 kHz. Signal areas are shown in arbitrary units. Abbrevations: Qa and Qb are the Cα peaks for the 'a' and 'b' conformers of the polyQ amyloid core; $PIIC_\alpha$ = Cα peak for polyproline II Pro residues; Prc = Cα peak for prolines in random coil; PC_δ = Cδ peak for all Pro residues (PP_{II} and random coil overlapping).

	Qa		Qb		$PIIC_\alpha$		PC_δ		Prc	
**Temp. (K)**	**linewidth (kHz)**	**signal area**	**linewidth (kHz)**	**signal area**	**linewidth (kHz)**	**signal area**	**linewidth (kHz)**	**signal area**	**linewidth (kHz)**	**signal area**
277	0.3	$1.2\cdot10^8$	0.2	$8.4\cdot10^7$	0.2	$4.3\cdot10^7$	0.2	$4.2\cdot10^7$	0.2	$2.4\cdot10^7$
270	0.3	$1.2\cdot10^8$	0.2	$8.9\cdot10^7$	0.2	$5.1\cdot10^7$	0.2	$4.8\cdot10^7$	0.3	$2.7\cdot10^7$
263	0.3	$1.1\cdot10^8$	0.3	$1.0\cdot10^8$	0.2	$6.0\cdot10^7$	0.2	$5.6\cdot10^7$	0.5	$4.9\cdot10^7$
256	0.3	$1.3\cdot10^8$	0.3	$1.2\cdot10^8$	0.3	$1.1\cdot10^8$	0.2	$8.4\cdot10^7$	0.3	$3.1\cdot10^7$

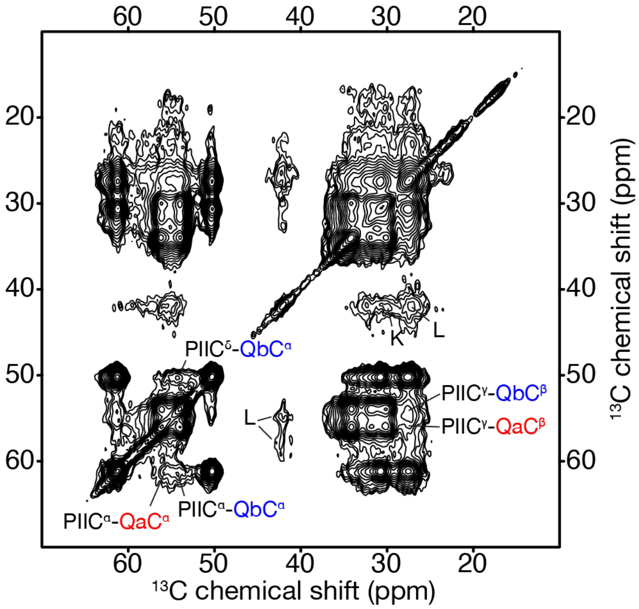

Figure S3. 2D ^{13}C-^{13}C PDSD experiment on Q44-HttEx1 fibrils at 256K. Long range magnetization transfer was performed using 500ms mixing time. Inter-residue correlations between prolines in PPII helix conformation in the C-terminus and type "a" and "b" glutamines are labeled.

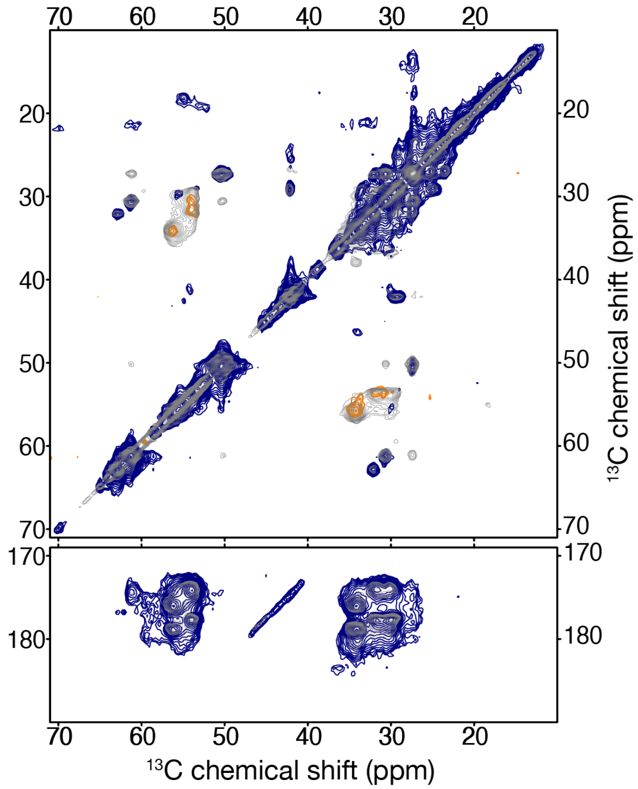

Figure S4. Overlay of filtered and unfiltered 2D ^{13}C-^{13}C spectra of Q44-HttEx1 fibrils. Grey spectrum represents the normal ^{13}C-^{13}C 2D DARR experiment (also shown in Figure 7A). The blue spectrum (with orange negative cross-peaks) shows the dynamically filtered ^{13}C-^{13}C DIPSHIFT-DARR 2D spectrum (Figure 7B). Both spectra were recorded at 277K with a DARR mixing time of 25ms.

Table S3. Overview of selected ^{13}C ssNMR chemical shifts of glutamines of Q44-HttEx1 and other polyQ fibrils. The uncertainty of chemical shifts is ±0.1-0.3ppm unless otherwise stated.

	**Chemical shifts (ppm)**					**Reference**
**Residue**	**C'**	**C_α**	**C_β**	**C_γ**	**C_δ**	**-**
Gln "a"	176.0	56.2	34.2	34.2	178.8	-
Gln "b"	174.0	54.0	31.7	30.0	177.7	-
Gln "c"	-	55.5	29.8	33.8	180.4	-
Gln "d"	173.6	53.3	29.0	29.0		-
Gln "c" published	-	-	-	33.9	180.3	(Hoop et al., 2016)
Q47c1	172.5	53.7	29.1	33.4	180.4	(Hoop et al., 2014)
Q47c2	173.0+0.5	53.0	30.3	34.3	178.6	(Hoop et al., 2014)
Q3	174.0	55.7	29.9	33.9	180.2	(Schneider et al., 2011)
Gln "a"	175.8	56.1	34.2	34.1	178.5	(Isas et al., 2015)
Gln "b"	173.9	54.0	31.8	29.9	177.4	(Isas et al., 2015)
Gln "c"	173.8	53.4	29.1	33.5	180.5	(Isas et al., 2015)

References cited in the Supporting Information

Hoop, C. L., Lin, H.-K., Kar, K., Hou, Z., Poirier, M. A., Wetzel, R., & Wel, P. C. A. van der. (2014). Polyglutamine Amyloid Core Boundaries and Flanking Domain Dynamics in Huntingtin Fragment Fibrils Determined by Solid-State Nuclear Magnetic Resonance. *Biochemistry*, *53*(42), 6653–6666. https://doi.org/10.1021/bi501010q

Hoop, C. L., Lin, H.-K., Kar, K., Magyarfalvi, G., Lamley, J. M., Boatz, J. C., Mandal, A., Lewandowski, J. R., Wetzel, R., & Wel, P. C. A. van der. (2016). Huntingtin exon 1 fibrils feature an interdigitated β-hairpin–based polyglutamine core. *Proceedings of the National Academy of Sciences*, *113*(6), 1546–1551. https://doi.org/10.1073/pnas.1521933113

Isas, J. M., Langen, R., & Siemer, A. B. (2015). Solid-State Nuclear Magnetic Resonance on the Static and Dynamic Domains of Huntingtin Exon-1 Fibrils. *Biochemistry*, *54*(25), 3942–3949. https://doi.org/10.1021/acs.biochem.5b00281

Lin, H.-K., Boatz, J. C., Krabbendam, I. E., Kodali, R., Hou, Z., Wetzel, R., Dolga, A. M., Poirier, M. A., & Wel, P. C. A. van der. (2017). Fibril polymorphism affects immobilized non-amyloid flanking domains of huntingtin exon1 rather than its polyglutamine core. *Nature Communications*, *8*, 15462. https://doi.org/10.1038/ncomms15462

Schneider, R., Schumacher, M. C., Mueller, H., Nand, D., Klaukien, V., Heise, H., Riedel, D., Wolf, G., Behrmann, E., Raunser, S., Seidel, R., Engelhard, M., & Baldus, M. (2011). Structural characterization of polyglutamine fibrils by solid-state NMR spectroscopy. *Journal of Molecular Biology*, *412*(1), 121–136. https://doi.org/10.1016/j.jmb.2011.06.045
